# Supplementary material for: Mirvetuximab Soravtansine in solid tumors: A systematic review and meta-analysis
Source: PLoS One. 2024 Dec 27;19(12):e0310736. doi: 10.1371/journal.pone.0310736 (PMC11676571; doi:10.1371/journal.pone.0310736)
Supplement: S1 Checklist — (DOCX) [file pone.0310736.s001.docx]

**Identification of studies via databases**

Records removed *before screening*:

Duplicate records removed (n = 289)

Not match the research direction (n= 61)

Records identified from databases:

PubMed (n= 41)

Embase (n= 315)

Cochrane Library (n= 27)

Web of science  (n= 110)

**Identification**

Records screened

(n = 143)

Records excluded for reasons:

Reviews (n = 102)

Reports excluded:

Pharmacology research

(n = 16)

Preclinical research

(n = 8)

Study protocol

(n = 8)

Reports assessed for eligibility

(n = 41)

**Screening**

Studies included in review

(n = 9)

Reports of included studies

(n = 10 )

**Included**
