# Supplementary material for: Mirvetuximab Soravtansine in solid tumors: A systematic review and meta-analysis
Source: PLoS One. 2024 Dec 27;19(12):e0310736. doi: 10.1371/journal.pone.0310736 (PMC11676571; doi:10.1371/journal.pone.0310736)
Supplement: S1 File — (DOC) [file pone.0310736.s005.doc]

### Mirvetuximab Soravtansine in Solid Tumors: a Systematic Review and Meta-Analysis ——Protocol

**Introduction:**

Mirvetuximab Soravtansine(MIRV) is a promising antibody-drug conjugate(ADC) that targets folate receptor alpha (FRα), which is overexpressed in several types of solid tumors. In November 2022, mirvetuximab soravtansine was approved in the USA for the treatment of adult patients with FRα positive, platinum-resistant epithelial ovarian, fallopian tube or primary peritoneal cancer who have received 1-3 prior systemic treatment regimens. Therefore , the high quality evidence for its efficacy and safety in different cancers is urgently needed. Meanwhile ,there been some early phase clinical trail has reported relevant result of its clinical activity for us to analysis.

**Objectives:**

This study aims to conduct a systematic review and meta-analysis to evaluate the efficacy and safety of MIRV, administered alone or in combination with chemo- and/or target-therapies in solid tumors. Potential differences in the drug efficacy and incidence of adverse events among cancer types, FRα expression level, prior treatment history, and drug combination,were also summarized to provide a reference for clinicians and patients in practice

**Methods:**

**Literature search:**

We will search (e.g. PubMed, Embase, Web Of Science, Cochrane Library)with the terms“mirvetuximab soravtansine” “IMGN853”“antibody-drug conjugate”, “ADC”, “solid tumor”, “cancer”to identify all relevant clinical trials of MIRV alone or in combination with chemo- and/or target-therapies in solid tumors, published from inception to August 29, 2026 The language will be restricted to English and Chinese. In addition, the reference lists of the selected articles were manually searched to identify additional studies.

**Eligibility criteria:**

Clinical trials of any design (single arm or randomized controlled trial) will be eligible for inclusion, if they study the use of MIRV alone or in combination with chemo- and/or target-therapies in any type of solid tumor

The exclusion criteria were: (1) abstracts of meetings; and (2) reviews and systematic reviews, meta-analyses, cost-effectiveness analyses, editorials, opinions or case reports

**Study selection:**

Two reviewers will independently evaluate all identified articles by title and abstract, and then review the full-text articles of potentially eligible studies. Disagreements will be resolved by a third reviewer. We will use the PRISMA flowchart to document the study selection process.

**Data extraction:**

Two reviewers will independently extract data from the included studies, using a pre-designed data extraction form. Data will be extracted on (1)study characteristics: first author, publication year, country of region, study design, sample size, intervention,(2) patient characteristics: tumor type, FRα expression level, prior treatment history/administrated alone or combined with chemo-therapies, (3)study outcomes: HR with 95 % CI for OS and PFS as primary endpoint and relative risk (RR) with 95 % CI for overall response rate (ORR) and adverse events (AEs) as secondary endpoint. Disagreements will be resolved by discuss with a third reviewer.

**Risk of bias assessment:**

Two reviewers will independently assess the quality and risk of bias of the included studies, using the Cochrane Risk of Bias tool for RCTs and the ROBINS-I tool for non-RCTs. Disagreements will be resolved by discuss with a third reviewer.

**Data analysis:**

All statistical analyses were conducted using Revman (version 5.3; Cochrane Collaboration Network) on homogeneous clinical studies. The heterogeneity was estimated by Cochran's Chi-square-based Q-test. The random effect model based on generic inverse variance method was applied when heterogeneity was significant (I2 > 50%). Subgroup analysis was performed according to tumor type, FRα expression level, prior treatment history/administrated alone or combined with chemo-therapies. In addition, sensitivity analysis was used to assess whether a single study dominated the results of the meta-analysis. Finally, the publication bias was evaluated by visual inspection of the funnel plots. All statistical tests were two-sided, and statistical significance was defined as P value less than 0.05. We will use the GRADE approach to assess the quality of the evidence.

**Ethical considerations:**

Ethical approval is not required for literature-based studies.
