# Supplementary material for: Mirvetuximab Soravtansine in solid tumors: A systematic review and meta-analysis
Source: PLoS One. 2024 Dec 27;19(12):e0310736. doi: 10.1371/journal.pone.0310736 (PMC11676571; doi:10.1371/journal.pone.0310736)
Supplement: S2 File — (DOCX) [file pone.0310736.s006.docx]

1. **Embase**

Books@Ovid <April 03, 2023>

Search All Ovid Journals@Ovid (abstracts only)

Journals@ovid Full Text of Sichuan University

EBM Reviews - ACP Journal Club <1991 to February 2023>

EBM Reviews - Cochrane Central Register of Controlled Trials <March 2023>

EBM Reviews - Cochrane Database of Systematic Reviews <2005 to April 4, 2023>

EBM Reviews - Cochrane Clinical Answers <March 2023>

EBM Reviews - Cochrane Methodology Register <3rd Quarter 2012>

EBM Reviews - Database of Abstracts of Reviews of Effects <1st Quarter 2016>

EBM Reviews - Health Technology Assessment <4th Quarter 2016>

EBM Reviews - NHS Economic Evaluation Database <1st Quarter 2016>

BIOSIS Previews <1995 to 2020>

Drug Information Full Text <March 2023>

Embase <1974 to 2023 April 07>

International Pharmaceutical Abstracts <1970 to March 2023>

Ovid MEDLINE(R) and Epub Ahead of Print, In-Process, In-Data-Review & Other Non-Indexed Citations, Daily and Versions <1946 to April 07, 2023>

1 solid tumor.mp. [mp=tx, bt, bo, ti, ot, ab, ct, sh, mc, st, or, tn, ps, ds, cb, rn, sq, mq, ge, tm, mi, kw, bc, cc, gn, fx, hw, on, sy, de, th, dm, mf, dv, kf, dq, rw, nm, ox, px, rx, an, ui, ux, mx] 134102

2 cancer.mp. [mp=tx, bt, bo, ti, ot, ab, ct, sh, mc, st, or, tn, ps, ds, cb, rn, sq, mq, ge, tm, mi, kw, bc, cc, gn, fx, hw, on, sy, de, th, dm, mf, dv, kf, dq, rw, nm, ox, px, rx, an, ui, ux, mx] 10806310

3 mirvetuximab soravtansine.mp. [mp=tx, bt, bo, ti, ot, ab, ct, sh, mc, st, or, tn, ps, ds, cb, rn, sq, mq, ge, tm, mi, kw, bc, cc, gn, fx, hw, on, sy, de, th, dm, mf, dv, kf, dq, rw, nm, ox, px, rx, an, ui, ux, mx] 407

4 IMGN853.mp. [mp=tx, bt, bo, ti, ot, ab, ct, sh, mc, st, or, tn, ps, ds, cb, rn, sq, mq, ge, tm, mi, kw, bc, cc, gn, fx, hw, on, sy, de, th, dm, mf, dv, kf, dq, rw, nm, ox, px, rx, an, ui, ux, mx] 158

5 antibody-drug conjugate.mp. [mp=tx, bt, bo, ti, ot, ab, ct, sh, mc, st, or, tn, ps, ds, cb, rn, sq, mq, ge, tm, mi, kw, bc, cc, gn, fx, hw, on, sy, de, th, dm, mf, dv, kf, dq, rw, nm, ox, px, rx, an, ui, ux, mx] 18651

6 ADC.mp. [mp=tx, bt, bo, ti, ot, ab, ct, sh, mc, st, or, tn, ps, ds, cb, rn, sq, mq, ge, tm, mi, kw, bc, cc, gn, fx, hw, on, sy, de, th, dm, mf, dv, kf, dq, rw, nm, ox, px, rx, an, ui, ux, mx] 82806

7 5 or 6 94267

8 3 or 4 461

9 1 or 2 10843332

10 7 and 8 329

11 9 and 10 315

<https://ovidsp.ovid.com/ovidweb.cgi?T=JS&NEWS=N&PAGE=main&SHAREDSEARCHID=9PtGz5OID0aw0OLxLmCEFXQduSst9kNOvQQSErherJpnM0tfot7TnLHjCllQek3V>

1. **# Web of Science 检索策略 (v0.1)**

# 数据库: 所有数据库

# 权限:

- WOS: 1900 to 2023

- DIIDW: 1966 to 2023

- INSPEC: 1969 to 2023

- KJD: 1980 to 2023

- MEDLINE: 1950 to 2023

- PPRN: 1991 to 2023

- SCIELO: 2002 to 2023

# 检索:

1: (TS=(mirvetuximab soravtansine)) OR TS=(IMGN853) and 预印本 (排除 – 数据库) 运行日期: Sun Apr 09 2023 21:07:35 GMT+0800 (CST) 检索结果: 180

2: ((TS=(Neoplasms)) OR TS=(cancer)) OR TS=(solid tumor) and 预印本 (排除 – 数据库) 运行日期: Sun Apr 09 2023 21:19:46 GMT+0800 (CST) 检索结果: 6835220

3: ((TS=(Immunoconjugates)) OR TS=(antibody-drug conjugate)) OR TS=(ADC) and 预印本 (排除 – 数据库) 运行日期: Sun Apr 09 2023 21:21:30 GMT+0800 (CST) 检索结果: 94162

4: #3 AND #1 and 预印本 (排除 – 数据库) 运行日期: Sun Apr 09 2023 21:24:06 GMT+0800 (CST) 检索结果: 115

5: #4 AND #2 and 预印本 (排除 – 数据库) 运行日期: Sun Apr 09 2023 21:25:14 GMT+0800 (CST) 检索结果: 110

1. **Pubmed**

Search number Query Sort By Filters Search Details Results Time

20 (((("Immunoconjugates"[Mesh]) OR ((antibody-drug conjugate))) OR (ADC)) AND ((("mirvetuximab soravtansine" [Supplementary Concept]) OR (IMGN853)) OR (mirvetuximab soravtansine))) AND ((("Neoplasms"[Mesh]) OR (solid tumor)) OR (cancer)) ("Immunoconjugates"[MeSH Terms] OR ("Immunoconjugates"[Pharmacological Action] OR "Immunoconjugates"[MeSH Terms] OR "Immunoconjugates"[All Fields] OR ("antibody"[All Fields] AND "drug"[All Fields] AND "conjugate"[All Fields]) OR "antibody drug conjugate"[All Fields]) OR ("arch dis child"[Journal] OR "acta dermatovenerol croat"[Journal] OR "adc"[All Fields])) AND ("mirvetuximab soravtansine"[Supplementary Concept] OR ("mirvetuximab soravtansine"[Supplementary Concept] OR "mirvetuximab soravtansine"[All Fields] OR "imgn853"[All Fields]) OR ("mirvetuximab soravtansine"[Supplementary Concept] OR "mirvetuximab soravtansine"[All Fields])) AND ("Neoplasms"[MeSH Terms] OR (("solid"[All Fields] OR "solid s"[All Fields] OR "solids"[All Fields]) AND ("cysts"[MeSH Terms] OR "cysts"[All Fields] OR "cyst"[All Fields] OR "neurofibroma"[MeSH Terms] OR "neurofibroma"[All Fields] OR "neurofibromas"[All Fields] OR "tumor s"[All Fields] OR "tumoral"[All Fields] OR "tumorous"[All Fields] OR "tumour"[All Fields] OR "Neoplasms"[MeSH Terms] OR "Neoplasms"[All Fields] OR "tumor"[All Fields] OR "tumour s"[All Fields] OR "tumoural"[All Fields] OR "tumourous"[All Fields] OR "tumours"[All Fields] OR "tumors"[All Fields])) OR ("cancer s"[All Fields] OR "cancerated"[All Fields] OR "canceration"[All Fields] OR "cancerization"[All Fields] OR "cancerized"[All Fields] OR "cancerous"[All Fields] OR "Neoplasms"[MeSH Terms] OR "Neoplasms"[All Fields] OR "cancer"[All Fields] OR "cancers"[All Fields])) 41 9:17:12

19 ((("Immunoconjugates"[Mesh]) OR ((antibody-drug conjugate))) OR (ADC)) AND ((("mirvetuximab soravtansine" [Supplementary Concept]) OR (IMGN853)) OR (mirvetuximab soravtansine)) ("Immunoconjugates"[MeSH Terms] OR ("Immunoconjugates"[Pharmacological Action] OR "Immunoconjugates"[MeSH Terms] OR "Immunoconjugates"[All Fields] OR ("antibody"[All Fields] AND "drug"[All Fields] AND "conjugate"[All Fields]) OR "antibody drug conjugate"[All Fields]) OR ("arch dis child"[Journal] OR "acta dermatovenerol croat"[Journal] OR "adc"[All Fields])) AND ("mirvetuximab soravtansine"[Supplementary Concept] OR ("mirvetuximab soravtansine"[Supplementary Concept] OR "mirvetuximab soravtansine"[All Fields] OR "imgn853"[All Fields]) OR ("mirvetuximab soravtansine"[Supplementary Concept] OR "mirvetuximab soravtansine"[All Fields])) 43 9:16:50

18 (("mirvetuximab soravtansine" [Supplementary Concept]) OR (IMGN853)) OR (mirvetuximab soravtansine) "mirvetuximab soravtansine"[Supplementary Concept] OR "mirvetuximab soravtansine"[Supplementary Concept] OR "mirvetuximab soravtansine"[All Fields] OR "imgn853"[All Fields] OR "mirvetuximab soravtansine"[Supplementary Concept] OR "mirvetuximab soravtansine"[All Fields] 56 8:59:28

16 (("Immunoconjugates"[Mesh]) OR ((antibody-drug conjugate))) OR (ADC) "Immunoconjugates"[MeSH Terms] OR ("Immunoconjugates"[Pharmacological Action] OR "Immunoconjugates"[MeSH Terms] OR "Immunoconjugates"[All Fields] OR ("antibody"[All Fields] AND "drug"[All Fields] AND "conjugate"[All Fields]) OR "antibody drug conjugate"[All Fields]) OR ("arch dis child"[Journal] OR "acta dermatovenerol croat"[Journal] OR "adc"[All Fields]) 57,790 8:58:43

15 (("Neoplasms"[Mesh]) OR (solid tumor)) OR (cancer) "Neoplasms"[MeSH Terms] OR (("solid"[All Fields] OR "solid s"[All Fields] OR "solids"[All Fields]) AND ("cysts"[MeSH Terms] OR "cysts"[All Fields] OR "cyst"[All Fields] OR "neurofibroma"[MeSH Terms] OR "neurofibroma"[All Fields] OR "neurofibromas"[All Fields] OR "tumor s"[All Fields] OR "tumoral"[All Fields] OR "tumorous"[All Fields] OR "tumour"[All Fields] OR "Neoplasms"[MeSH Terms] OR "Neoplasms"[All Fields] OR "tumor"[All Fields] OR "tumour s"[All Fields] OR "tumoural"[All Fields] OR "tumourous"[All Fields] OR "tumours"[All Fields] OR "tumors"[All Fields])) OR ("cancer s"[All Fields] OR "cancerated"[All Fields] OR "canceration"[All Fields] OR "cancerization"[All Fields] OR "cancerized"[All Fields] OR "cancerous"[All Fields] OR "Neoplasms"[MeSH Terms] OR "Neoplasms"[All Fields] OR "cancer"[All Fields] OR "cancers"[All Fields]) 4,844,699 8:58:24

14 cancer "cancer s"[All Fields] OR "cancerated"[All Fields] OR "canceration"[All Fields] OR "cancerization"[All Fields] OR "cancerized"[All Fields] OR "cancerous"[All Fields] OR "neoplasms"[MeSH Terms] OR "neoplasms"[All Fields] OR "cancer"[All Fields] OR "cancers"[All Fields] 4,834,099 8:58:08

13 solid tumor ("solid"[All Fields] OR "solid s"[All Fields] OR "solids"[All Fields]) AND ("cysts"[MeSH Terms] OR "cysts"[All Fields] OR "cyst"[All Fields] OR "neurofibroma"[MeSH Terms] OR "neurofibroma"[All Fields] OR "neurofibromas"[All Fields] OR "tumor s"[All Fields] OR "tumoral"[All Fields] OR "tumorous"[All Fields] OR "tumour"[All Fields] OR "neoplasms"[MeSH Terms] OR "neoplasms"[All Fields] OR "tumor"[All Fields] OR "tumour s"[All Fields] OR "tumoural"[All Fields] OR "tumourous"[All Fields] OR "tumours"[All Fields] OR "tumors"[All Fields]) 118,289 8:57:32

12 ADC "arch dis child"[Journal] OR "acta dermatovenerol croat"[Journal] OR "adc"[All Fields] 40,991 8:57:21

11 (antibody-drug conjugate) "immunoconjugates"[Pharmacological Action] OR "immunoconjugates"[MeSH Terms] OR "immunoconjugates"[All Fields] OR ("antibody"[All Fields] AND "drug"[All Fields] AND "conjugate"[All Fields]) OR "antibody drug conjugate"[All Fields] 18,563 8:57:13

8 IMGN853 "mirvetuximab soravtansine"[Supplementary Concept] OR "mirvetuximab soravtansine"[All Fields] OR "imgn853"[All Fields] 56 8:55:51

7 mirvetuximab soravtansine "mirvetuximab soravtansine"[Supplementary Concept] OR "mirvetuximab soravtansine"[All Fields] 53 8:55:35

5 "Immunoconjugates"[Mesh] Most Recent "Immunoconjugates"[MeSH Terms] 13,583 8:51:31

4 "mirvetuximab soravtansine" [Supplementary Concept] Most Recent "mirvetuximab soravtansine"[Supplementary Concept] 29 8:51:08

3 "Neoplasms"[Mesh] Most Recent "Neoplasms"[MeSH Terms] 3,813,576 8:50:29

**4.Central**

PMID Title Authors Citation First Author Journal/Book Publication Year Create Date PMCID NIHMS ID DOI

33667670 Phase III, randomized trial of mirvetuximab soravtansine versus chemotherapy in patients with platinum-resistant ovarian cancer: primary analysis of FORWARD I Moore KN, Oza AM, Colombo N, Oaknin A, Scambia G, Lorusso D, Konecny GE, Banerjee S, Murphy CG, Tanyi JL, Hirte H, Konner JA, Lim PC, Prasad-Hayes M, Monk BJ, Pautier P, Wang J, Berkenblit A, Vergote I, Birrer MJ. Ann Oncol. 2021 Jun;32(6):757-765. doi: 10.1016/j.annonc.2021.02.017. Epub 2021 Mar 2. Moore KN Ann Oncol 2021 2021/3/5 10.1016/j.annonc.2021.02.017

36472472 Antibodies to watch in 2023 Kaplon H, Crescioli S, Chenoweth A, Visweswaraiah J, Reichert JM. MAbs. 2023 Jan-Dec;15(1):2153410. doi: 10.1080/19420862.2022.2153410. Kaplon H MAbs 2023 2022/12/6 PMC9728470 10.1080/19420862.2022.2153410

32152484 Exploiting the folate receptor α in oncology Scaranti M, Cojocaru E, Banerjee S, Banerji U. Nat Rev Clin Oncol. 2020 Jun;17(6):349-359. doi: 10.1038/s41571-020-0339-5. Epub 2020 Mar 9. Scaranti M Nat Rev Clin Oncol 2020 2020/3/11 10.1038/s41571-020-0339-5

28029313 Safety and Activity of Mirvetuximab Soravtansine (IMGN853), a Folate Receptor Alpha-Targeting Antibody-Drug Conjugate, in Platinum-Resistant Ovarian, Fallopian Tube, or Primary Peritoneal Cancer: A Phase I Expansion Study Moore KN, Martin LP, O'Malley DM, Matulonis UA, Konner JA, Perez RP, Bauer TM, Ruiz-Soto R, Birrer MJ. J Clin Oncol. 2017 Apr 1;35(10):1112-1118. doi: 10.1200/JCO.2016.69.9538. Epub 2016 Dec 28. Moore KN J Clin Oncol 2017 2016/12/29 PMC5559878 10.1200/JCO.2016.69.9538

25904506 IMGN853, a Folate Receptor-α (FRα)-Targeting Antibody-Drug Conjugate, Exhibits Potent Targeted Antitumor Activity against FRα-Expressing Tumors Ab O, Whiteman KR, Bartle LM, Sun X, Singh R, Tavares D, LaBelle A, Payne G, Lutz RJ, Pinkas J, Goldmacher VS, Chittenden T, Lambert JM. Mol Cancer Ther. 2015 Jul;14(7):1605-13. doi: 10.1158/1535-7163.MCT-14-1095. Epub 2015 Apr 22. Ab O Mol Cancer Ther 2015 2015/4/24 10.1158/1535-7163.MCT-14-1095

32081463 Phase Ib study of mirvetuximab soravtansine, a folate receptor alpha (FRα)-targeting antibody-drug conjugate (ADC), in combination with bevacizumab in patients with platinum-resistant ovarian cancer O'Malley DM, Matulonis UA, Birrer MJ, Castro CM, Gilbert L, Vergote I, Martin LP, Mantia-Smaldone GM, Martin AG, Bratos R, Penson RT, Malek K, Moore KN. Gynecol Oncol. 2020 May;157(2):379-385. doi: 10.1016/j.ygyno.2020.01.037. Epub 2020 Feb 18. O'Malley DM Gynecol Oncol 2020 2020/2/22 10.1016/j.ygyno.2020.01.037

29098867 A review of mirvetuximab soravtansine in the treatment of platinum-resistant ovarian cancer Moore KN, Martin LP, O'Malley DM, Matulonis UA, Konner JA, Vergote I, Ponte JF, Birrer MJ. Future Oncol. 2018 Jan;14(2):123-136. doi: 10.2217/fon-2017-0379. Epub 2017 Nov 3. Moore KN Future Oncol 2018 2017/11/4 10.2217/fon-2017-0379

36656533 Mirvetuximab Soravtansine: First Approval Heo YA. Drugs. 2023 Feb;83(3):265-273. doi: 10.1007/s40265-023-01834-3. Heo YA Drugs 2023 2023/1/19 10.1007/s40265-023-01834-3

30531606 Antibody-drug conjugates for ovarian cancer: current clinical development Stewart D, Cristea M. Curr Opin Obstet Gynecol. 2019 Feb;31(1):18-23. doi: 10.1097/GCO.0000000000000515. Stewart D Curr Opin Obstet Gynecol 2019 2018/12/12 10.1097/GCO.0000000000000515

32618744 Novel antibody-drug conjugates: current and future roles in gynecologic oncology Tymon-Rosario J, Zeybek B, Santin AD. Curr Opin Obstet Gynecol. 2021 Feb 1;33(1):26-33. doi: 10.1097/GCO.0000000000000642. Tymon-Rosario J Curr Opin Obstet Gynecol 2021 2020/7/4 PMC8253558 NIHMS1602375 10.1097/GCO.0000000000000642

30379722 Ocular Toxicity of Mirvetuximab Corbelli E, Miserocchi E, Marchese A, Giuffrè C, Berchicci L, Sacconi R, Bandello F, Modorati GM. Cornea. 2019 Feb;38(2):229-232. doi: 10.1097/ICO.0000000000001805. Corbelli E Cornea 2019 2018/11/1 10.1097/ICO.0000000000001805

28843653 Characterization of folate receptor alpha (FRα) expression in archival tumor and biopsy samples from relapsed epithelial ovarian cancer patients: A phase I expansion study of the FRα-targeting antibody-drug conjugate mirvetuximab soravtansine Martin LP, Konner JA, Moore KN, Seward SM, Matulonis UA, Perez RP, Su Y, Berkenblit A, Ruiz-Soto R, Birrer MJ. Gynecol Oncol. 2017 Nov;147(2):402-407. doi: 10.1016/j.ygyno.2017.08.015. Epub 2017 Aug 24. Martin LP Gynecol Oncol 2017 2017/8/28 PMC6893864 NIHMS1060350 10.1016/j.ygyno.2017.08.015

30093227 Safety and activity findings from a phase 1b escalation study of mirvetuximab soravtansine, a folate receptor alpha (FRα)-targeting antibody-drug conjugate (ADC), in combination with carboplatin in patients with platinum-sensitive ovarian cancer Moore KN, O'Malley DM, Vergote I, Martin LP, Gonzalez-Martin A, Malek K, Birrer MJ. Gynecol Oncol. 2018 Oct;151(1):46-52. doi: 10.1016/j.ygyno.2018.07.017. Epub 2018 Aug 6. Moore KN Gynecol Oncol 2018 2018/8/11 10.1016/j.ygyno.2018.07.017

26136852 Vintafolide: a novel targeted therapy for the treatment of folate receptor expressing tumors Vergote I, Leamon CP. Ther Adv Med Oncol. 2015 Jul;7(4):206-18. doi: 10.1177/1758834015584763. Vergote I Ther Adv Med Oncol 2015 2015/7/3 PMC4480526 10.1177/1758834015584763

36046840 Advancing antibody-drug conjugates in gynecological malignancies: myth or reality? Nerone M, Grande MD, Sessa C, Colombo I. Explor Target Antitumor Ther. 2022;3(2):149-171. doi: 10.37349/etat.2022.00077. Epub 2022 Apr 19. Nerone M Explor Target Antitumor Ther 2022 2022/9/1 PMC9400759 10.37349/etat.2022.00077

28534292 Development and Characterization of a Neutralizing Anti-idiotype Antibody Against Mirvetuximab for Analysis of Clinical Samples Loebrich S, Shen M, Cohen E, Payne G, Chen Y, Bogalhas M, Zhao Y. AAPS J. 2017 Jul;19(4):1223-1234. doi: 10.1208/s12248-017-0098-0. Epub 2017 May 22. Loebrich S AAPS J 2017 2017/5/24 10.1208/s12248-017-0098-0

33812984 Revisiting antibody-drug conjugates and their predictive biomarkers in platinum-resistant ovarian cancer El Bairi K, Al Jarroudi O, Afqir S. Semin Cancer Biol. 2021 Dec;77:42-55. doi: 10.1016/j.semcancer.2021.03.031. Epub 2021 Apr 1. El Bairi K Semin Cancer Biol 2021 2021/4/4 10.1016/j.semcancer.2021.03.031

30929824 Antibody-drug conjugates in gynecologic malignancies Lee EK, Liu JF. Gynecol Oncol. 2019 Jun;153(3):694-702. doi: 10.1016/j.ygyno.2019.03.245. Epub 2019 Mar 28. Lee EK Gynecol Oncol 2019 2019/4/2 10.1016/j.ygyno.2019.03.245

29116596 Antibody-Drug Conjugates for the Treatment of Solid Tumors: Clinical Experience and Latest Developments Nagayama A, Ellisen LW, Chabner B, Bardia A. Target Oncol. 2017 Dec;12(6):719-739. doi: 10.1007/s11523-017-0535-0. Nagayama A Target Oncol 2017 2017/11/9 10.1007/s11523-017-0535-0

32984932 A phase II study of Mirvetuximab Soravtansine in triple-negative breast cancer Yam C, Rauch GM, Rahman T, Karuturi M, Ravenberg E, White J, Clayborn A, McCarthy P, Abouharb S, Lim B, Litton JK, Ramirez DL, Saleem S, Stec J, Symmans WF, Huo L, Damodaran S, Sun R, Moulder SL. Invest New Drugs. 2021 Apr;39(2):509-515. doi: 10.1007/s10637-020-00995-2. Epub 2020 Sep 28. Yam C Invest New Drugs 2021 2020/9/28 10.1007/s10637-020-00995-2

27889646 Mirvetuximab Soravtansine (IMGN853), a Folate Receptor Alpha-Targeting Antibody-Drug Conjugate, Potentiates the Activity of Standard of Care Therapeutics in Ovarian Cancer Models Ponte JF, Ab O, Lanieri L, Lee J, Coccia J, Bartle LM, Themeles M, Zhou Y, Pinkas J, Ruiz-Soto R. Neoplasia. 2016 Dec;18(12):775-784. doi: 10.1016/j.neo.2016.11.002. Epub 2016 Nov 25. Ponte JF Neoplasia 2016 2016/11/28 PMC5126132 10.1016/j.neo.2016.11.002

36716407 Efficacy and Safety of Mirvetuximab Soravtansine in Patients With Platinum-Resistant Ovarian Cancer With High Folate Receptor Alpha Expression: Results From the SORAYA Study Matulonis UA, Lorusso D, Oaknin A, Pignata S, Dean A, Denys H, Colombo N, Van Gorp T, Konner JA, Marin MR, Harter P, Murphy CG, Wang J, Noble E, Esteves B, Method M, Coleman RL. J Clin Oncol. 2023 Jan 30:JCO2201900. doi: 10.1200/JCO.22.01900. Online ahead of print. Matulonis UA J Clin Oncol 2023 2023/1/30 10.1200/JCO.22.01900

36736157 Safety and efficacy of mirvetuximab soravtansine, a folate receptor alpha (FRα)-targeting antibody-drug conjugate (ADC), in combination with bevacizumab in patients with platinum-resistant ovarian cancer Gilbert L, Oaknin A, Matulonis UA, Mantia-Smaldone GM, Lim PC, Castro CM, Provencher D, Memarzadeh S, Method M, Wang J, Moore KN, O'Malley DM. Gynecol Oncol. 2023 Mar;170:241-247. doi: 10.1016/j.ygyno.2023.01.020. Epub 2023 Feb 1. Gilbert L Gynecol Oncol 2023 2023/2/3 10.1016/j.ygyno.2023.01.020

28440955 Phase 1 dose-escalation study of mirvetuximab soravtansine (IMGN853), a folate receptor α-targeting antibody-drug conjugate, in patients with solid tumors Moore KN, Borghaei H, O'Malley DM, Jeong W, Seward SM, Bauer TM, Perez RP, Matulonis UA, Running KL, Zhang X, Ponte JF, Ruiz-Soto R, Birrer MJ. Cancer. 2017 Aug 15;123(16):3080-3087. doi: 10.1002/cncr.30736. Epub 2017 Apr 25. Moore KN Cancer 2017 2017/4/26 PMC6896318 NIHMS1060533 10.1002/cncr.30736

34683998 Antibody-Antineoplastic Conjugates in Gynecological Malignancies: Current Status and Future Perspectives Martín-Sabroso C, Lozza I, Torres-Suárez AI, Fraguas-Sánchez AI. Pharmaceutics. 2021 Oct 15;13(10):1705. doi: 10.3390/pharmaceutics13101705. Martín-Sabroso C Pharmaceutics 2021 2021/10/23 PMC8541375 10.3390/pharmaceutics13101705

29440294 In Vitro and In Vivo Activity of IMGN853, an Antibody-Drug Conjugate Targeting Folate Receptor Alpha Linked to DM4, in Biologically Aggressive Endometrial Cancers Altwerger G, Bonazzoli E, Bellone S, Egawa-Takata T, Menderes G, Pettinella F, Bianchi A, Riccio F, Feinberg J, Zammataro L, Han C, Yadav G, Dugan K, Morneault A, Ponte JF, Buza N, Hui P, Wong S, Litkouhi B, Ratner E, Silasi DA, Huang GS, Azodi M, Schwartz PE, Santin AD. Mol Cancer Ther. 2018 May;17(5):1003-1011. doi: 10.1158/1535-7163.MCT-17-0930. Epub 2018 Feb 13. Altwerger G Mol Cancer Ther 2018 2018/2/15 PMC5932245 NIHMS936296 10.1158/1535-7163.MCT-17-0930

32463296 Antibody-drug conjugates for the treatment of ovarian cancer Calo CA, O'Malley DM. Expert Opin Biol Ther. 2021 Jul;21(7):875-887. doi: 10.1080/14712598.2020.1776253. Epub 2020 Jun 8. Calo CA Expert Opin Biol Ther 2021 2020/5/29 10.1080/14712598.2020.1776253

30413525 Evaluation of Prophylactic Corticosteroid Eye Drop Use in the Management of Corneal Abnormalities Induced by the Antibody-Drug Conjugate Mirvetuximab Soravtansine Matulonis UA, Birrer MJ, O'Malley DM, Moore KN, Konner J, Gilbert L, Martin LP, Bauer TM, Oza AM, Malek K, Pinkas J, Kim SK. Clin Cancer Res. 2019 Mar 15;25(6):1727-1736. doi: 10.1158/1078-0432.CCR-18-2474. Epub 2018 Nov 9. Matulonis UA Clin Cancer Res 2019 2018/11/11 10.1158/1078-0432.CCR-18-2474

29424243 FORWARD I: a Phase III study of mirvetuximab soravtansine versus chemotherapy in platinum-resistant ovarian cancer Moore KN, Vergote I, Oaknin A, Colombo N, Banerjee S, Oza A, Pautier P, Malek K, Birrer MJ. Future Oncol. 2018 Jul;14(17):1669-1678. doi: 10.2217/fon-2017-0646. Epub 2018 Feb 9. Moore KN Future Oncol 2018 2018/2/10 10.2217/fon-2017-0646

31369274 Folate Receptor α-Targeted (89)Zr-M9346A Immuno-PET for Image-Guided Intervention with Mirvetuximab Soravtansine in Triple-Negative Breast Cancer Heo GS, Detering L, Luehmann HP, Primeau T, Lee YS, Laforest R, Li S, Stec J, Lim KH, Lockhart AC, Liu Y. Mol Pharm. 2019 Sep 3;16(9):3996-4006. doi: 10.1021/acs.molpharmaceut.9b00653. Epub 2019 Aug 16. Heo GS Mol Pharm 2019 2019/8/2 10.1021/acs.molpharmaceut.9b00653

30155674 Leveraging PET to image folate receptor α therapy of an antibody-drug conjugate Brand C, Sadique A, Houghton JL, Gangangari K, Ponte JF, Lewis JS, Pillarsetty NVK, Konner JA, Reiner T. EJNMMI Res. 2018 Aug 28;8(1):87. doi: 10.1186/s13550-018-0437-x. Brand C EJNMMI Res 2018 2018/8/30 PMC6113196 10.1186/s13550-018-0437-x

30585545 Clinical Trials of Novel Targeted Therapies in Ovarian Cancer: Moving Beyond Poly ADP Ribose Polymerase (PARP) Inhibitors Guo Q, Yang Q, Li J, Liu G, Nikoulin I, Jia S. Curr Pharm Biotechnol. 2018;19(14):1114-1121. doi: 10.2174/1389201020666181226123054. Guo Q Curr Pharm Biotechnol 2018 2018/12/27 10.2174/1389201020666181226123054

32674207 Mirvetuximab Soravtansine Combination Yields Encouraging Response Rates in Ovarian Cancer Fowler M. Oncology (Williston Park). 2020 Jul 15;34(7):250. Fowler M Oncology (Williston Park) 2020 2020/7/17

36618922 A review of recent advances on single use of antibody-drug conjugates or combination with tumor immunology therapy for gynecologic cancer Wang AJ, Gao Y, Shi YY, Dai MY, Cai HB. Front Pharmacol. 2022 Dec 22;13:1093666. doi: 10.3389/fphar.2022.1093666. eCollection 2022. Wang AJ Front Pharmacol 2022 2023/1/9 PMC9813853 10.3389/fphar.2022.1093666

36878560 Integrating antibody drug conjugates in the management of gynecologic cancers Chelariu-Raicu A, Mahner S, Moore KN, Lorusso D, Coleman RL. Int J Gynecol Cancer. 2023 Mar 6;33(3):420-429. doi: 10.1136/ijgc-2022-003701. Chelariu-Raicu A Int J Gynecol Cancer 2023 2023/3/6 10.1136/ijgc-2022-003701

34140289 "Significant Activity" for ADC in Ovarian Cancer Cancer Discov. 2021 Aug;11(8):OF3. doi: 10.1158/2159-8290.CD-NB2021-0361. Epub 2021 Jun 17. Cancer Discov 2021 2021/6/18 10.1158/2159-8290.CD-NB2021-0361

31098752 Known and novel ocular toxicities of biologics, targeted agents, and traditional chemotherapeutics Kunkler AL, Binkley EM, Mantopoulos D, Hendershot AJ, Ohr MP, Kendra KL, Davidorf FH, Cebulla CM. Graefes Arch Clin Exp Ophthalmol. 2019 Aug;257(8):1771-1781. doi: 10.1007/s00417-019-04337-8. Epub 2019 May 16. Kunkler AL Graefes Arch Clin Exp Ophthalmol 2019 2019/5/18 10.1007/s00417-019-04337-8

37023499 The evolving landscape of antibody-drug conjugates in gynecologic cancers Tolcher A, Hamilton E, Coleman RL. Cancer Treat Rev. 2023 Mar 20;116:102546. doi: 10.1016/j.ctrv.2023.102546. Online ahead of print. Tolcher A Cancer Treat Rev 2023 2023/4/6 10.1016/j.ctrv.2023.102546

27797594 Folate receptor alpha antagonists in preclinical and early stage clinical development for the treatment of epithelial ovarian cancer Bergamini A, Ferrero S, Leone Roberti Maggiore U, Scala C, Pella F, Vellone VG, Petrone M, Rabaiotti E, Cioffi R, Candiani M, Mangili G. Expert Opin Investig Drugs. 2016 Dec;25(12):1405-1412. doi: 10.1080/13543784.2016.1254616. Bergamini A Expert Opin Investig Drugs 2016 2016/11/1 10.1080/13543784.2016.1254616

34955159 Prise en charge médicale de la récidive du cancer épithélial de l'ovaire: Medical management of recurrent epithelial ovarian cancer Pautier P, Motte-Rouge T, Lécuru F, Classe JM, Ferron G, Floquet A, Kurtz JE, Freyer G, Hardy-Bessard AC. Bull Cancer. 2021 Dec;108(9S1):S22-S32. doi: 10.1016/S0007-4551(21)00584-1. Pautier P Bull Cancer 2021 2021/12/27 10.1016/S0007-4551(21)00584-1

30659135 Highlights of the NCCN Oncology Research Program J Natl Compr Canc Netw. 2019 Jan;17(1):xxxvii. doi: 10.6004/jnccn.2019.0005. J Natl Compr Canc Netw 2019 2019/1/20 10.6004/jnccn.2019.0005
