# Supplementary material for: Mirvetuximab Soravtansine in solid tumors: A systematic review and meta-analysis
Source: PLoS One. 2024 Dec 27;19(12):e0310736. doi: 10.1371/journal.pone.0310736 (PMC11676571; doi:10.1371/journal.pone.0310736)
Supplement: S6 File — (DOCX) [file pone.0310736.s010.docx]

**Explanation of how missing data were handled**

Dear Editors,

In our submitted Mirvetuximab Soravtansine in Solid Tumors: A Systematic Review and Meta-Analysis, we would like to provide a detailed account of how missing data were addressed during the study process.

1. Exclusion of Studies with Incomplete Data During Literature Search:

At the initial stage of our literature review, we implemented a stringent selection process to exclude studies with incomplete data. This was done to ensure that only studies with comprehensive and reliable information were included in our meta-analysis. By rigorously screening the literature, we were able to maintain a high standard of data quality and integrity.

1. Statistical Methods Employed to Address Missing Data in Meta-analysis:**
2. Subgroup Analysis:

We conducted subgroup analyses to explore potential sources of heterogeneity and to account for differences in study characteristics that could be related to missing data. This approach helped us to identify and control for variations in the data that might have been influenced by missing information.

1. Sensitivity Analysis:

To assess the robustness of our findings, we performed sensitivity analyses under different assumptions about the missing data. This included evaluating the impact of assuming that the data were missing completely at random (MCAR) or missing at random (MAR). These analyses provided us with confidence in the stability of our results despite the presence of missing data.

1. Mixed Effects Models:

Recognizing the potential hierarchical structure of our data, we utilized mixed effects models in our meta-statistical procedures. This allowed us to estimate both fixed and random effects, thereby accommodating the missing data and providing a more accurate reflection of the treatment effect in the context of the studies included in our meta-analysis.

By employing these methods, we aimed to minimize the potential biases and errors that could arise from missing data, ensuring that our conclusions regarding the efficacy and safety of Mirv in solid tumors are as accurate and reliable as possible.

We are confident that our approach to handling missing data is in line with best practices in meta-analysis and believe that it enhances the validity of our findings.
