# Supplementary material for: Mirvetuximab Soravtansine in solid tumors: A systematic review and meta-analysis
Source: PLoS One. 2024 Dec 27;19(12):e0310736. doi: 10.1371/journal.pone.0310736 (PMC11676571; doi:10.1371/journal.pone.0310736)
Supplement: S7 File — (DOCX) [file pone.0310736.s011.docx]

1. **Primary end-point: mPFS**

| **firstauthor drug** | **mpfs** | **ll** | **ul** | **n** | **mono/combine** | **Title** |
| --- | --- | --- | --- | --- | --- | --- |
| **Moore 2021** | **9.6** | **5.4** | **14.1** | **248** | **mono** | **Phase III, randomized trial of mirvetuximab soravtansine versus chemotherapy in patients with platinum-resistant ovarian cancer: primary analysis of FORWARD I** |
| **O'Malley 2019** | **6.9** | **4.9** | **8.6** | **66** | **combine** | **Phase Ib study of mirvetuximab soravtansine, a folate receptor alpha (FRα)-targeting antibody-drug conjugate (ADC), in combination with bevacizumab in patients with platinum-resistant ovarian cancer** |
| **Martina 2017** | **4.2** | **2.8** | **5.4** | **27** | **mono** | **Characterization of folate receptor alpha (FRα) expression in archival tumor and biopsy samples from relapsed epithelial ovarian cancer patients: A phase I expansion study of the FRα-targeting antibody-drug conjugate mirvetuximab soravtansine** |
| **Gilbert 2023** | **8.2** | **6.8** | **10** | **94** | **combine** | **Safety and efficacy of mirvetuximab soravtansine, a folate receptor alpha (FRα)-targeting antibody-drug conjugate (ADC), in combination with bevacizumab in patients with platinum-resistant ovarian cancer** |
| **Matulonis 2023** | **4.3** | **3.7** | **5.2** | **106** | **mono** | **Efficacy and Safety of Mirvetuximab Soravtansine in Patients With Platinum-Resistant Ovarian Cancer With High Folate Receptor Alpha Expression: Results From the SORAYA Study.** |
| **Backes 2021** | **6.3** | **0.7** | **13.8** | **21** | **combine** | **Phase i study of mirvetuximab soravtansine (MIRV) and rucaparib for recurrent endometrial, ovarian, fallopian tube or primary peritoneal cancer** |
| **Moore 2018** | **15** | **9.9** | **22** | **17** | **combine** | **Safety and activity findings from a phase 1b escalation study of mirvetuximab soravtansine, a folate receptor alpha (FRα)-targeting antibody-drug conjugate (ADC), in combination with carboplatin in patients with platinum-sensitive ovarian cancer** |
| **O’Malley 2020** | **12** | **9** | **15** | **9** | **combine** | **Mirvetuximab soravtansine (MIRV), a folate receptor alpha (FR[alpha])-targeting antibody-drug conjugate (ADC), in combination with carboplatin (CARBO) and bevacizumab (BEV): Final results from a study in patients (pts) with recurrent platinum sensitive ovarian cancer: 833P.** |
| **Moore 2017** | **4.8** | **3.9** | **5.7** | **46** | **mono** | **Safety and Activity of Mirvetuximab Soravtansine (IMGN853), a Folate Receptor Alpha-Targeting Antibody-Drug Conjugate, in Platinum-Resistant Ovarian, Fallopian Tube, or Primary Peritoneal Cancer: A Phase I Expansion Study** |

1. **Primary end-point: ORR**

| **Study ID** | **ORR** | **LL** | **UL** | **N** | **Title** |  |
| --- | --- | --- | --- | --- | --- | --- |
| **Moore 2021** | **0.22** | **0.0276** | **0.1534** | **243** | **Phase III, randomized trial of mirvetuximab soravtansine versus chemotherapy in patients with platinum-resistant ovarian cancer: primary analysis of FORWARD I** |  |
| **O'Malley 2019** | **0.56** | **0.3** | **0.8** | **16** | **Phase Ib study of mirvetuximab soravtansine, a folate receptor alpha (FRα)-targeting antibody-drug conjugate (ADC), in combination with bevacizumab in patients with platinum-resistant ovarian cancer** |  |
| **Martina 2017** | **0.258** | **0** | **1** | **21** | **Characterization of folate receptor alpha (FRα) expression in archival tumor and biopsy samples from relapsed epithelial ovarian cancer patients: A phase I expansion study of the FRα-targeting antibody-drug conjugate mirvetuximab soravtansine** |  |
| **Gilbert 2023** | **0.446** | **0.368** | **0.541** | **83** | **Safety and efficacy of mirvetuximab soravtansine, a folate receptor alpha (FRα)-targeting antibody-drug conjugate (ADC), in combination with bevacizumab in patients with platinum-resistant ovarian cancer** |  |
| **Matulonis 2023** | **0.353** | **0.224** | **0.499** | **51** | **Efficacy and Safety of Mirvetuximab Soravtansine in Patients With Platinum-Resistant Ovarian Cancer With High Folate Receptor Alpha Expression: Results From the SORAYA Study.** |  |
| **Moore 2018** | **0.71** | **0.3688** | **1.0431** | **17** | **Phase i study of mirvetuximab soravtansine (MIRV) and rucaparib for recurrent endometrial, ovarian, fallopian tube or primary peritoneal cancer** |  |
| **O’Malley 2020** | **0.39** | **0.4318** | **0.75** | **66** | **Safety and activity findings from a phase 1b escalation study of mirvetuximab soravtansine, a folate receptor alpha (FRα)-targeting antibody-drug conjugate (ADC), in combination with carboplatin in patients with platinum-sensitive ovarian cancer** |  |
| **Moore 2017** | **0.222** | **0.028** | **0.6** | **9** | **Mirvetuximab soravtansine (MIRV), a folate receptor alpha (FR[alpha])-targeting antibody-drug conjugate (ADC), in combination with carboplatin (CARBO) and bevacizumab (BEV): Final results from a study in patients (pts) with recurrent platinum sensitive ovarian cancer: 833P.** |  |
| **Mihaela 2021** | **0.36** | **0.2192** | **0.7142** | **30** | **Safety and Activity of Mirvetuximab Soravtansine (IMGN853), a Folate Receptor Alpha-Targeting Antibody-Drug Conjugate, in Platinum-Resistant Ovarian, Fallopian Tube, or Primary Peritoneal Cancer: A Phase I Expansion Study** |  |

1. **Subgroup analysis**

| **Study ID** | **ORR** | **LL** | **UL** | **N** | **Subgroup** |
| --- | --- | --- | --- | --- | --- |
| **Gilbert 2023** | **0.446** | **0.368** | **0.541** | **83** | **High FRα expression** |
| **O'Malley 2020** | **0.56** | **0.3** | **0.8** | **16** | **High FRα expression** |
| **Moore 2018** | **0.8** | **0.553** | **1.047** | **10** | **High FRα expression** |
| **Martina 2017** | **0.258** | **0** | **1** | **21** | **High FRα expression** |
| **Moore 2017** | **0.266** | **0.2645** | **0.2675** | **37** | **High FRα expression** |
|  |  |  |  |  |  |
| **Gilbert 2023** | **0.36** | **0.11** | **0.69** | **11** | **Low FRα expression** |
| **Moore 2017** | **0.222** | **0.028** | **0.6** | **9** | **Low FRα expression** |
|  |  |  |  |  |  |
| **Gilbert 2023** | **0.44** | **0.3366** | **0.5997** | **94** | **Platinum resistant** |
| **Matulonis 2023** | **0.324** | **0.212** | **0.426** | **105** | **Platinum resistant** |
| **Mihaela 2021** | **0.36** | **0.2192** | **0.7142** | **30** | **Platinum resistant** |
| **Moore 2021** | **0.22** | **0.0276** | **0.1534** | **243** | **Platinum resistant** |
| **O'Malley 2020** | **0.39** | **0.4318** | **0.75** | **66** | **Platinum resistant** |
| **Moore 2017** | **0.26** | **0.0806** | **0.3976** | **46** | **Platinum resistant** |
|  |  |  |  |  |  |
| **Gilbert 2023** | **0.48** | **0.3039** | **0.7928** | **31** | **Platinum sensetive** |
| **Moore 2018** | **0.71** | **0.3688** | **1.0431** | **17** | **platinum sensitive** |
|  |  |  |  |  |  |
| **Matulonis 2023** | **0.302** | **0.183** | **0.443** | **53** | **≧3 lines of Prior therapy** |
| **O'Malley 2020** | **0.39** | **0.239** | **0.541** | **39** | **≧3 lines of Prior therapy** |
|  |  |  |  |  |  |
| **Matulonis 2023** | **0.353** | **0.224** | **0.499** | **51** | **1～2 lines of Prior therapy** |
| **O'Malley 2020** | **0.56** | **0.3** | **0.8** | **16** | **1～2 lines of Prior therapy** |
|  |  |  |  |  |  |
| **Matulonis 2023** | **0.324** | **0.2165** | **0.4315** | **105** | **MIRV** |
| **Moore 2021** | **0.22** | **0.1578** | **0.2822** | **243** | **MIRV** |
| **Moore 2017** | **0.26** | **0.1011** | **0.4189** | **46** | **MIRV** |
| **Martina 2017** | **0.222** | **0.076** | **0.368** | **27** | **MIRV** |
|  |  |  |  |  |  |
| **Gilbert 2023** | **0.44** | **0.3084** | **0.5716** | **94** | **MIRV + BEV** |
| **Gilbert 2023** | **0.48** | **0.2346** | **0.7254** | **31** | **MIRV + BEV** |
| **O'Malley 2020** | **0.39** | **0.2308** | **0.5492** | **66** | **MIRV + BEV** |

1. **All grades dverse effects**

| **firstauthor** | **n** | **nausea** | **diarrhea** | **fatigue** | **blurred vision** | **Dry eye** | **thrombocytopenia** | **keratopathy** | **neuropathy** | **Vomiting** | **Decreased appetite** | **Headache** | **ALT increased** | **AST increased** | **Pneumonia** | **anemia** |
| --- | --- | --- | --- | --- | --- | --- | --- | --- | --- | --- | --- | --- | --- | --- | --- | --- |
| **Moore 2021** | **243** | **0.46** | **0.31** | **0.29** | **0.42** | **0.26** | **0.10** | **0.33** | **0.27** | **0.16** | **0.17** |  |  | **0.17** |  | **0.11** |
| **O'Malley 2019** | **66** | **0.46** | **0.52** | **0.41** | **0.50** | **0.27** | **0.30** | **0.24** | **0.35** | **0.24** | **0.26** | **0.24** | **0.24** | **0.26** |  |  |
| **Martina 2017** | **27** | **0.27** | **0.37** | **0.44** | **0.37** | **0.15** |  | **0.48** | **0.26** |  | **0.11** | **0.19** | **0.22** | **0.26** | **0.15** |  |
| **Gilbert 2023** | **94** | **0.51** | **0.54** | **0.43** | **0.57** | **0.28** | **0.30** | **0.34** | **0.38** | **0.27** | **0.28** | **0.27** | **0.23** | **0.27** |  |  |
| **Matulonis 2023** | **106** | **0.29** | **0.22** | **0.24** | **0.41** | **0.25** |  | **0.29** | **0.13** | **0.11** | **0.13** |  |  |  |  |  |
| **Moore 2017** | **46** | **0.37** | **0.44** | **0.30** | **0.41** | **0.13** |  | **0.26** | **0.28** | **0.22** |  | **0.11** | **0.15** | **0.24** |  | **0.13** |
| **Moore 2018** | **18** | **0.67** | **0.61** | **0.56** | **0.61** |  | **0.61** | **0.22** | **0.44** | **0.50** | **0.33** | **0.28** | **0.22** | **0.22** | **0.50** | **0.39** |
| **Backes 2021** | **21** | **0.67** |  | **0.73** | **0.60** | **0.33** | **0.27** |  |  | **0.27** |  |  | **0.40** | **0.40** |  | **0.47** |
| **O’Malley 2020** | **41** | **0.76** | **0.83** | **0.76** | **0.68** |  |  |  |  |  |  |  |  |  |  |  |

1. **≧3 grades dverse effects**

| **firstauthor** | **n** | **nausea** | **diarrhea** | **fatigue** | **blurred vision** | **Dry eye** | **thrombocytopenia** | **keratopathy** | **neuropathy** | **Vomiting** | **Decreased appetite** | **Headache** | **ALT increased** | **AST increased** | **pneumonitis** | **anemia** |
| --- | --- | --- | --- | --- | --- | --- | --- | --- | --- | --- | --- | --- | --- | --- | --- | --- |
| **Moore 2021** | **243** | **0.012** | **0.021** | **0.012** | **0.025** | **0.012** |  | **0.012** | **0.025** | **0.012** | **0.008** |  |  | **0.012** |  | **0.008** |
| **O'Malley 2019** | **66** | **0.015** | **0.015** | **0.015** | **0.015** | **0.015** | **0.045** |  |  | **0.015** |  |  | **0.045** | **0.061** |  |  |
| **Martina 2017** | **27** |  |  |  |  |  |  |  |  |  |  |  |  |  |  |  |
| **Gilbert 2023** | **94** | **0.01** | **0.01** | **0.03** | **0.01** | **0.02** | **0.04** |  | **0.01** | **0.01** |  |  | **0.03** | **0.04** |  |  |
| **Matulonis 2023** | **106** |  | **0.02** | **0.01** | **0.06** | **0.02** |  | **0.09** |  |  | **0.01** |  |  |  |  |  |
| **Backes 2021** | **21** |  | **0.07** | **0.2** |  |  | **0.07** |  |  |  |  |  |  |  | **0.13** | **0.13** |
| **Moore 2018** | **18** |  | **0.056** | **0.056** |  |  | **0.167** |  |  |  |  |  |  |  |  | **0.111** |
| **O’Malley 2020** | **41** | **0.02** | **0.1** | **0.05** | **0.1** |  |  |  |  |  |  |  |  |  |  |  |
| **Moore 2017** | **46** | **0.022** | **0.022** | **0.043** |  |  |  |  | **0.022** | **0.022** |  |  | **0.022** | **0.022** |  | **0.022** |

| **AEs** | **Any grade** |  |
| --- | --- | --- |
|  | **ES, % (95 % CI)** | **I2, %** |
| **Vision blurred** | **45.20(39.32-51.09)** | **45.18** |
| **Nausea** | **40.13 (32.27-47.99)** | **70.57** |
| **Diarrhea** | **39.52 (28.86-50.18)** | **84.98** |
| **Fatigue** | **33.84 (26.90-40.77)** | **64.25** |
| **Keratopathy** | **31.2 (27.45-34.94)** | **0.03** |
| **Neuropathy** | **27.33 (19.46-35.19)** | **76.61** |
| **AST increased** | **23.58 (18.29-28.87)** | **36.60** |
| **Dry eye** | **23.30(18.70-27.90)** | **37.45** |
| **Thrombocytopenia** | **23.17 (12.01-34.34]** | **82.59** |
| **ALT increased** | **22.19(17.29-27.09)** | **0.01** |
| **Headache** | **20.29(12.52-28.06)** | **54.79** |
| **Vomiting** | **19.54(13.79-25.28)** | **62.74** |
| **Decreased appetite** | **18.71(12.58-24.85)** | **66.80** |
| **Anemia** | **12.96(9.41-16.51)** | **81.08** |
