# Supplementary material for: Mirvetuximab Soravtansine in solid tumors: A systematic review and meta-analysis
Source: PLoS One. 2024 Dec 27;19(12):e0310736. doi: 10.1371/journal.pone.0310736 (PMC11676571; doi:10.1371/journal.pone.0310736)
Supplement: Attachment — Submitted filename: Response to Reviewer.docx [file pone.0310736.s012.docx]

**Response to Reviewer**

**Dear editor,**

**Thank you for the opportunity to revise our manuscript, “Mirvetuximab Soravtansine in Solid Tumors: A Systematic Review and Meta-Analysis”, which we submitted to PLOS ONE. We appreciate the feedback from the reviewers and have carefully considered their comments. Below, we provide a point-by-point response to the reviewers' comments and have highlighted the changes made in the revised manuscript.**

**Reviewer #1: The systematic review and meta-analysis by Rehim et al, provide a much needed analysis of the therapeutic benefits of MIRV based on several important variables including prior treatments, platinum response status, monotherapy and combination therapy, and FR-alpha expression levels, as well as adverse drug effects. This type of review is essential for identifying how all these variables affect therapeutic efficacy and safety and what research direction is needed to address outstanding therapeutic concerns. A major limitation of the current study is that many of the studies are not randomized controlled trials and the authors have duly acknowledged this limitation which is not of their own making. Nonetheless, the findings are very interesting. However, I do have several suggestions that will help improve the quality of the manuscript:**

**Comment #1:** The data on prior lines of treatment is quite interesting but it would be helpful to further stratify the prior line of treatments based on the actual treatments the patients where receiving, their platinum response status, and their FR-alpha expression levels. This will help eliminate any potential confounding factors preventing us from seeing the importance of prior lines of treatment to the therapeutic effects of MIRV.

**Response:** We agree with the reviewer that stratifying the data based on specific treatments, platinum response status, and FR-alpha expression levels would provide a more nuanced understanding of the impact of prior lines of treatment on the therapeutic effects of Mirvetuximab Soravtansine (MIRV). Following your suggestion, I have further stratified the patients’ actual systemic chemotherapy treatments, drug types exposed during treatment, platinum response status, and FR-α expression levels in the table of main characteristics of the included studies[Table 1]. Unfortunately, only a very small number of studies provided effect estimates based on these subgroups, and no study provided survival data for each patient. Therefore, in our previous statistical analysis, we only conducted subgroup analyses using the data that were available, including patients’ platinum responsiveness, the number of previous systemic chemotherapies, and FR-α expression levels. Indeed, through these subgroup analyses, we were able to observe that these factors do have some impact on the clinical benefits of the drug. The specific analyses are presented in Figures [Figure4,5].

**Comment #2**: For table 1, the age should be provided as median IQR, and for the number of prior systemic treatments, what does the n mean? You provide a number and then another number in brackets. That is unclear.

**Response:**We apologize for the lack of clarity in Table 1. We agree that presenting the age as median with the interquartile range (IQR) would be more informative. However, it is regrettable that all the original studies only provided the median age and range, and we were unable to obtain individual patient age data for statistical processing. Regarding the “No. of prior systemic treatments,” the three numbers provided represent the number of prior systemic chemotherapies the patients received, the number of patients who received that number of treatments, and the percentage of these patients within the total study population, respectively. We have updated the table legend to include this explanation, which we hope will provide readers with a clearer understanding.

**Comment #3:** It is important to spell out words in full at first mention and then continue using that abbreviation onwards. This is not addressed for PRISMA, HR, RECIST, CTCAE, AE.

**Response:** We acknowledge this oversight and have now ensured that all terms are spelled out in full at their first mention in the manuscript, followed by the corresponding abbreviation, as per the journal’s guidelines. We have corrected this throughout the text to maintain consistency and clarity.

**Comment #4**: There is definitely something wrong with the referencing method used in this paper, it is very inconsistent and unclear. The authors are advised to address this issue.

**Response：**We agree that the referencing style in the initial submission was inconsistent and have now standardized the referencing throughout the manuscript to follow the PLOS ONE citation guidelines. We have checked each reference for accuracy and formatting consistency, and we are confident that the revised manuscript adheres to the journal’s standards.

**In addition to addressing the specific comments from the reviewers, we have made several other improvements to the manuscript. In Table 1, we have now included the chemotherapy agents to which patients were previously exposed and the FR-α expression levels of the patients in each study. This additional information provides a more comprehensive overview of the patient population and their treatment history, which we believe will enhance the understanding of the study outcomes.**

**We hope that the revisions we have made address the concerns raised by the reviewers and improve the quality of our manuscript. We are confident that our work is of significant interest to the readers of Plos one and we thank you for considering our work for publication.Please do not hesitate to contact us if you require any further information.**

**Sincerely,**

**Dr. Hongjing Wang**

Organization: Key Laboratory of Birth Defects and Related Diseases of Women and Children of the Ministry of Education, West China Second University Hospital, Sichuan University, Chengdu, China

Email address: [whjscdx@163.com](mailto:whjscdx@163.com)
